# Effect of Support Matrix and Crosslinking Agents on Nutritional Properties of Orange Juice during Enzyme Clarification: A Comparative Study

**DOI:** 10.3390/foods12213919

**Published:** 2023-10-26

**Authors:** Pâmela M. da Silva, Eli Emanuel Esparza-Flores, Anike H. Virgili, Eliana W. de Menezes, Roberto Fernandez-Lafuente, Lucas Dal Magro, Rafael C. Rodrigues

**Affiliations:** 1 Biocatalysis and Enzyme Technology Laboratory, Food Science and Technology Institute, Federal University of Rio Grande do Sul, 9500 Bento Gonçalves Avenue, P.O. Box 15090, Porto Alegre 91501-970, RS, Brazil; pamelamnutri@gmail.com (P.M.d.S.); eli.esparza.f@gmail.com (E.E.E.-F.); 2 LSS—Laboratory of Solids and Surfaces, Instituto de Química, Federal University of Rio Grande do Sul, Porto Alegre 91501-970, RS, Brazil; anikehv@gmail.com (A.H.V.); eliana.weber@ufrgs.br (E.W.d.M.); 3 Departamento de Biocatálisis, Instituto de Catálisis-CSIC, Campus UAM-CSIC Madrid, 28049 Madrid, Spain; rfl@icp.csic.es; 4 Instituto Federal de Educação Ciência e Tecnologia Sul-Rio-Grandense—IFSul, Pelotas 96015-360, RS, Brazil; lucas.dalmagro@yahoo.com.br

**Keywords:** pectinase, enzyme immobilization, orange juice, clarification, chitosan, silica, genipin, glutaraldehyde, nutritional properties

## Abstract

This study investigated the impact of a support matrix and active group on the support to the nutritional properties of orange juice after juice clarification. Pectinase was immobilized on chitosan and aminated silica supports, activated with genipin or glutaraldehyde, and applied for juice clarification. The effects on various juice properties, including reducing sugars, total soluble solids, vitamin C, and phenolic compounds, juice color, and pH, were evaluated. The results revealed that the immobilization on chitosan activated using genipin resulted in the highest biocatalyst activity (1211.21 U·g^−1^). The juice treatments using the biocatalysts led to turbidity reduction in the juice (up to 90%), with the highest reductions observed in treatments involving immobilized enzyme on chitosan. Importantly, the enzymatic treatments preserved the natural sugar content, total soluble solids, and pH of the juice. Color differences between treated and raw juice samples were especially relevant for those treated using enzymes, with significant differences in L* and b*, showing loss of yellow vivid color. Analysis of phenolic compounds and vitamin C showed no significant alterations after the enzymatic treatment of the raw juice. According to our results, the clarification of orange juice using immobilized enzymes can be a compromise in turbidity reduction and color reduction to maintain juice quality.

## 1. Introduction

Orange juice is a popular and widely consumed fruit juice due to its high nutritional value and health benefits [1,2]. It is a good source of vitamins, particularly vitamin C, which plays a crucial role in various physiological processes, such as collagen synthesis, immune function, and antioxidant protection [3]. In addition, orange juice is rich in phenolic compounds, which have been associated with a wide range of health benefits, such as antioxidant, anti-inflammatory, and anticancer properties [4,5,6]. Given the importance of orange juice as a nutritional beverage, it is essential to maintain its quality and nutritional value during processing and storage [3,7].

During its processing, orange juice undergoes clarification to remove suspended particles and impurities, which can affect its appearance, stability, and sensory attributes [8,9]. The clarification of fruit juices can be performed via membrane technology, using special ultrafiltration (UF) membranes to eliminate yeast, molds, microorganisms, colloids, as well as insoluble proteins, tannins, and polysaccharides. Additionally, these membranes are used to concentrate the juice [10,11,12,13]. Nonetheless, UF encounters the challenge of fouling. The fouling of the membrane leads to a reduction in flux and product rejection during operation, thereby resulting in elevated energy consumption [14]. The use of enzymes, such as pectinases, has been established as an effective method for the clarification of orange juice [8,15]. Through adding pectinases, the viscosity decreases and turbid particles are precipitated, making it easier to eliminate them through filtration or centrifugation. This enhances the yield of clarified juice and prolongs the lifespan of equipment such as filters [16]. However, the use of free enzymes in juice processing has limitations, such as enzyme degradation, reduced activity, and low reusability. To overcome these challenges, enzyme immobilization has been proposed as a promising approach for enzyme stabilization and reuse. The immobilization of enzymes can improve their stability through diverse mechanisms (as recently reviewed in [17], activity and selectivity, in addition to reducing enzyme degradation and loss during juice processing [18].

In this context, the choice of an adequate support matrix is a crucial step in this process, as it directly affects the efficiency and stability of the immobilized enzyme [19]. Chitosan and silica are two commonly used support matrices for enzyme immobilization in juice processing [20,21,22,23,24]. Chitosan, a biopolymer derived from chitin, has been shown to be an effective support matrix due to its biocompatibility, biodegradability, and ability to be activated with different reactive moieties able to form stable bonds with enzymes [25,26]. Silica, on the other hand, is an inorganic support matrix widely used due to its high surface area and porosity, which allows for efficient enzyme immobilization, mainly after its amination [27,28,29,30].

The covalent immobilization of enzymes on support matrices requires the use of a activating agent to covalently attach the enzyme to the matrix [31,32]. Glutaraldehyde is a widely used crosslinking agent that forms a covalent bond between the enzyme and support matrix through reacting with the amino groups on the enzyme surface [33]. Glutaraldehyde has been shown to be effective in stabilizing enzymes, improving their activity and allowing for reuse of the immobilized enzyme [34]. However, glutaraldehyde has also been associated with toxicity and environmental concerns, which limits its use in some applications [35]. In a recent study by Hosseine et al. [36], an alternative support activating agent was proposed for the immobilization of pectinases. They synthesized a polyaldehyde pullulan through the oxidation of pullulan. While they propose its potential application as an alternative to glutaraldehyde for enzyme immobilization, the control of carbohydrate oxidation may be a challenge.

On the other hand, genipin is a natural iridoid used as crosslinking agent derived from the fruit of Genipa americana [32]. Genipin forms covalent bonds with the amino groups of enzymes through nucleophilic addition, resulting in stable crosslinking [37]. This unique reaction mechanism allows genipin to maintain enzyme activity and stability [38], with fewer concerns about toxicity and environmental impact compared to glutaraldehyde. Despite these advantages, genipin is more expensive than glutaraldehyde, which may limit its use in large-scale industrial applications, usually and mainly for biocatalysts used in biomedicine [32].

Previous studies have been reported the successful use of immobilized enzymes for juice clarification, including pectinase immobilized on chitosan and silica supports [23,24]. However, these studies focused mainly on the clarification potential of the biocatalyst, without considering its effects on other important juice properties, such as possible alterations of nutritional content, color, and taste. Therefore, it is important to investigate the effect of immobilized enzymes on these juice properties to fully understand the potential benefits of this approach for juice processing. To the best of our knowledge, there is currently no published literature addressing the effect of immobilized biocatalysts on the sensory and nutritional properties of orange juice following clarification.

In this sense, we aimed to investigate the effect of a support matrix and support activating agent on the nutritional properties of orange juice during enzyme clarification. Specifically, we immobilized pectinase on chitosan and silica supports activated with either genipin or glutaraldehyde and applied these biocatalytic particles to orange juice clarification. We then evaluated the effect of these immobilized enzymes on juice clarification, as well as on other properties of the juice, including concentrations of reducing sugars, total soluble solids, vitamin C, phenolic compounds, and also color and pH. The results of this study will provide valuable insights into the development of improved enzyme immobilization techniques for juice processing, with the potential to improve both the quality and nutritional value of orange juice.

## 2. Materials and Methods

### 2.1. Materials

Freshly pressed orange juice without any additional treatment was kindly donated by Hugo Pietro (Caxias do Sul, RS, Brazil). Pectinex Ultra SP-L was kindly donated by Novozymes (Madrid, Spain). Pectin from apple, galacturonic acid, chitosan (from shrimp shells, ≥75% deacetylated), tetraethylorthosilicate 98% (TEOS), and 3-aminopropyltrimethoxysilane 97% (APTMS) were acquired from Sigma-Aldrich (St. Louis, MO, USA). Genipin from *Genipa americana* was obtained through the biphasic extraction method [39]. All other reagents and solvents were of analytical grade.

The results presented in this study represent the mean of three experiments, along with their respective standard deviations. Whenever applicable, the data were analyzed using analysis of variance (ANOVA) followed by Tukey’s test for multiple comparisons.

### 2.2. Pectinase Activity

Total pectinase activity was measured using pectin as the substrate, based on Dal Magro et al. [40]. For this, 0.1 mL of diluted enzyme was added to 0.9 mL of 1 g·L^−1^ of pectin in 50 mM sodium citrate at pH 4.8. The reaction mixture was incubated at 37 °C, for 1 min, under agitation. The amount of reducing groups was estimated via the 3,5-dinitrosalicyclic acid (DNS) method according to Miller [41] using galacturonic acid as standard. One unit of pectinase activity was defined as the amount of enzyme needed to produce 1 µmol of reducing groups per min under the reaction conditions.

### 2.3. Support Preparation

#### 2.3.1. Chitosan Beads

Chitosan particles were prepared using the precipitation method, according to Klein et al. [42]. A solution of chitosan (2% *w*/*v*) dissolved in 0.35 M acetic acid was sonicated for 30 min to remove the air bubbles. Then, it was dripped in 1 M sodium hydroxide (coagulation solution) under slow mechanical stirring. The formed particles, named CH, were separated via filtration, washed with distilled water until neutrality was reached, and stored for activation.

#### 2.3.2. Silica Particles

Silica was prepared through a sol–gel method utilizing tetraethylorthosilicate (TEOS) hydrolysis and condensation in the presence of HCl as a catalyst for the development of silanol groups on the support [43]. The synthesis procedure involved mixing 5 mL of TEOS, 5 mL of ethanol, and a mixture of 36 drops of HF/HCl (6/6 M) in 2 mL of solution. HCl was then added, leading to almost instantaneous gelation. The mixture was left to sit at room temperature for 14 days. Subsequently, the silica was ground using a mortar and pestle, washed with water and ethanol, and vacuum-dried at 90 °C for 2 h. The prepared silica was organofunctionalized with APTMS via the following process: the silica was heat-treated in a vacuum at 140 °C for 4 h, prior to organofunctionalization using 1 mmol of APTMS per gram of silica. The reaction was carried out in toluene at 80 °C with mechanical stirring under an inert atmosphere for 24 h. After the reaction, the supernatant was removed, and the materials were washed with toluene, ethanol, and water. Finally, the matrix, named S, was dried under vacuum at 80 °C for 2 h.

### 2.4. Support Activation and Enzyme Immobilization

#### 2.4.1. Support Activation with Glutaraldehyde

Both chitosan and silica particles were activated with glutaraldehyde using the procedure described by Dal Magro et al. [44]: 50 chitosan beads (20 mg dry mass) or 100 mg of silica were incubated in 0.1 M phosphate–potassium at pH 7.0 with 1% (*v*/*v*) glutaraldehyde at 37 °C under gentle stirring for 2 h. After, the supports, named CH-GLU and S-GLU, were recovered via filtration, washed 3 times with 50 mM sodium citrate at pH 4.8 to remove excess glutaraldehyde, and used for enzyme immobilization.

#### 2.4.2. Support Activation with Genipin

In the same way, both particles were activated with genipin following the protocol described by Flores et al. [45]: 50 chitosan beads (20 mg dry mass) or 100 mg of silica were added to genipin solution (1.5 mg·mL^−1^ in 0.1 M phosphate buffer at pH 9.0) and the reaction was performed at 60 °C for 1 h under gentle stirring. After, the supports, named CH-GEN and S-GEN, were recovered, washed 3 times with sodium citrate buffer (50 mM, pH 4.8) to remove excess genipin, and used for enzyme immobilization.

#### 2.4.3. Enzyme Immobilization on the Activated Supports

To prepare the immobilization of the enzyme, the genipin or glutaraldehyde activated supports were added to enzyme solutions previously diluted to 1:10 (m:v) in 50 mM sodium citrate buffer at pH 4.8 and room temperature in a roller mixer overnight. At the end, the biocatalysts were recovered and washed 3 times with 50 mM sodium citrate buffer (at pH 4.8 to remove the non-bound enzymes. Finally, the different biocatalysts (CH-GEN-E, CH-GLU-E, S-GEN-E, S-GLU-E) were suspended in 50 mM sodium citrate buffer at pH 4.8 and stored at 4 °C. The immobilization yield (IY), immobilization efficiency (IE), and recovered activity (RA) were calculated according to Sheldon and Van Pelt [46,47], following the equations:

(1) $$IY\left(\%\right)=\frac{Immobilized\;activity\;(U)}{Initial\;activity\;(U)}\times 100$$

(2) $$IE\left(\%\right)=\frac{Biocatalyst\;activity\;(U)}{Immobilized\;activity\;(U)}\times 100$$

(3) $$RA\left(\%\right)=\frac{Biocatalyst\;activity\;(U)}{Initial\;activity\;(U)}\times 100$$

The immobilized activity refers to the difference in activity observed between the initial free enzyme solution and the activity determined in the supernatant and washing fractions. The initial activity corresponds to the enzyme activity in its free form, before mixing to the support material. The biocatalyst activity is the activity measured on the support material after immobilization.

### 2.5. Juice Clarification

The orange juice clarification was carried out as reported by Dal Magro et al. [48], considering the clarification in terms of turbidity reduction, with 100% being the turbidity of the juice without any treatment. Briefly, 5 U of pectinase (CH-GEN-E, CH-GLU-E, S-GEN-E, S-GLU-E) was added to 1 mL of raw orange juice and incubated at 40 °C for 1 h. Additionally, in order to measure the matrix effect, non-activated particles (CH or S) and activated particles without enzymes (CH-GEN, CH-GLU, S-GEN, S-GLU) were also used in the clarification process.

### 2.6. Analytical Determinations

#### 2.6.1. Turbidity

Juice turbidity was measured using spectrophotometric analysis, based on light scattering, as proposed by Anderson [49], with some modifications. At the end of clarification, support particles were recovered via centrifugation for 2 min at 5000× *g*. Subsequently, the supernatants were analyzed using a spectrophotometer at 860 nm. The percentage of reduction in juice turbidity was calculated considering the absorbance of the control (juice without any treatment) and the absorbance of the treated samples.

#### 2.6.2. Total Soluble Solids (°Brix), Reducing Sugars, and pH of the Juice

Soluble solids (°Brix) were measured using a refractometer at 20.0 ± 0.5 °C. The reducing sugars were quantified using the DNS method, proposed by Miller [41]. The pH of the juice was measured using a digital pH meter.

#### 2.6.3. Color Determination

The determination of juice color was performed in a Minolta Colorimeter (Model CR-400, Konica Minolta Sensing, Inc., Osaka, Japan). It was based on 3 color coordinates: L* (whiteness or brightness/darkness), a* (redness/greenness), and b* (yellowness/blueness). The Chroma (C*) was calculated through following Equation (4):

(4) $$\mathrm{Chroma}\;(C^{*})=\sqrt{{\left(a^{*}\right)}^{2}+{\left(b^{*}\right)}^{2}}$$

#### 2.6.4. Ascorbic Acid Analysis via HPLC-DAD

The quantification of acid ascorbic acid was performed in HPLC using the method proposed by Osturk et al. [50] with some modifications. The analysis was performed using a Waters HPLC 2695 series system (Wilmington, NC, USA, EUA) coupled with a diode array detector, the Waters DAD 2998 (Wilmington, NC, USA, EUA), and arranged to 254 nm wavelengths. In the HPLC system, separation was achieved using the selective Aminex column (HPX-87H, 300 mm × 7.8 mm, particle size 9 μm, Bio-Rad Lab., Richmond, CA, USA). The mobile phase was 0.009 N H_2_SO_4_, filtered through a 0.45 μm filter.

#### 2.6.5. HPLC-DAD Analysis of Phenolic Compounds

Phenolic compound determination was performed using a Shimadzu high-performance liquid chromatographer (HPLC) (Kyoto, Japan), connected in series to a DAD detector (SPD-M20A). The chromatographic separation conditions were previously described by Rodrigues et al. [51]. Phenolic compounds were separated in a Synergi Hydro-RP column (250 × 4.6 mm, 4 μm, Phenomenex, Torrance, CA, USA) at 0.7 mL·min^−1^ at 35 °C, using a linear gradient of water:formic acid (99.9:0.1, *v*/*v*, solvent A) and acetonitrile:formic acid (99.9:0.1, *v*/*v*, solvent B) as the mobile phase. The UV-vis spectra were obtained between 200 and 800 nm, and the chromatograms were processed at 280, 320, and 360 nm.

## 3. Results and Discussion

### 3.1. Enzyme Immobilization

Initially, pectinase was immobilized on both supports, chitosan and silica, using both activating agents, the most used crosslinking agent, glutaraldehyde [33], and the less toxic alternative, genipin [52]. The results for the immobilization parameters are presented in Table 1.

Analyzing the results presented in Table 1, it is possible to observe that the biocatalysts immobilized using genipin presented higher activity than those immobilized using glutaraldehyde. For the pectinase immobilization on cationic PS resin activated with glutaraldehyde, Miao et al. [53] found around 550 U·g^−1^. The mechanism of immobilization is similar to the one used for chitosan activated with glutaraldehyde in our work: the modification of a cationic amino surface with glutaraldehyde and immobilization via Schiff’s base formation with amino groups between glutaraldehyde and the enzyme. The immobilization yield represents the efficiency of the immobilization process in terms of the proportion of the initial activity that is immobilized. The immobilization on chitosan–glutaraldehyde shows the highest immobilization yield. This could be attributed to the chemical properties of glutaraldehyde, which is known for its effectiveness in binding biomolecules by multifunctional factors [33]. Using silica activated with amino groups, Muller et al. [54] found a 7.7% immobilization yield. On the other hand, the same support showed the highest immobilization efficiency compared to chitosan. These differences might be attributed to the enzyme loading and the generation of substrate diffusional limitations, considering the large size of this molecule: the higher the enzyme load, the higher the effects of diffusional limitations on enzyme activity. Navarro-López et al. [55] immobilized pectinase on chitosan–magnetic nanoparticles coupled with glutaraldehyde and found 65.6% efficiency. Although the authors used chitosan-based support, the mechanism was similar to that used for silica in our work: functionalization with 3-APTMS and coupling with glutaraldehyde. The immobilization efficiency was similar for our study (57.6%). Concerning the support activating agents, genipin is relatively stable under physiological conditions and provides long-term stability to the cross-linked products [45], while glutaraldehyde is a highly reactive compound that rapidly reacts with biomolecules. Both are widely used for enzyme immobilization presenting different results depending on the biocatalyst, the immobilization conditions, and the nature of the support.

### 3.2. Juice Clarification

In order to investigate the effects of the support matrix, activating agent, and enzyme on the properties of the orange juice after enzymatic clarification, the chitosan and silica particles were applied to juice clarification. The particles were used as prepared, after activation or after enzyme immobilization. The results for turbidity reduction after clarification are presented in Table 2. When naked chitosan or silica were used for juice clarification, the results of turbidity reduction were very low, as well as for the activated particles without an enzyme.

The presence of enzymes in the particles led to a significant turbidity reduction for both chitosan and silica biocatalysts. Pectinase is responsible to breaking down the pectin and removing some of the turbidity-causing components in the orange juice [56]. Interestingly, the results indicated that there is no matrix effect on turbidity reduction. This suggests that the support materials (chitosan and silica) and activating agents (genipin and glutaraldehyde) primarily act as carriers for the enzyme, providing a suitable environment for its activity rather than directly contributing to the turbidity reduction process. The absence of a matrix effect implies that the immobilized enzyme retains its ability to clarify the juice, regardless of the support material or activating agent used. This finding reinforces the interest of utilizing immobilized enzymes for juice clarification.

When comparing both supports, the enzyme immobilized on chitosan exhibited slightly higher turbidity reduction compared to the enzyme immobilized on silica, around 8%, considering the mean of the biocatalysts. This observation is consistent with previous studies where chitosan-immobilized enzymes [48] demonstrated greater efficiency than silica-immobilized enzymes [23]. In a multi-enzymatic system composed of pectinase and protease immobilized on chitosan particles, the authors observed that the immediate reduction in turbidity after the enzymatic treatment (49%) was lower than that found in the present study. However, they noted a gradual reduction in turbidity over time, reaching 70% after 21 days of storage [24].

### 3.3. Total Soluble Solids, Reducing Sugars, and Color Parameters

The results of the treated juice for its physicochemical analysis are presented in Table 2. The results showed relatively minor variations in juice TSS, reducing sugars, and pH values among the different biocatalyst combinations. Although the results for TSS and reducing sugars presented statistical differences, there is no correlation considering the supports, activating agent, or presence of enzymes. This suggests that the clarification process, regardless of the support nature or crosslinking agent used, did not significantly alter the sweetness, sugar content, or acidity of the juice. This is positive, as it is desired that the clarification treatment does not affect the nutritional and sensorial parameters of the juice. Using a pectinase immobilized on glass beads, Azimi et al. [57] found a small reduction in TSS after clarification. The authors attributed this to the deposition of suspended compounds with pectin hydrolysis [57].

Regarding the color parameters, most of the treated juice presented similar values compared to raw orange juice. The main changes are in the L*, a*, and b* parameters in the treatments using enzymes which are statistically different from the rest of treatments and raw juice. It can be related to the effects of the enzymatic clarification process and turbidity reduction. The L* parameter represents the lightness of the sample, where higher values indicate a brighter appearance [58]. Comparing the treatments using enzymes to the raw orange juice, it can be observed that the L* values generally increased in the treatments. This suggests that the enzymatic clarification process, and the consequent turbidity reduction, contributed to a brighter appearance of the juice. On the other hand, the decrease in Chroma* values for the samples treated with enzymes indicates that the clarified juice presents a less vivid color [59]. This could be attributed to the decrease in the b* parameter, as presented in Table 2.

The b* parameter represents the position between yellow and blue, with positive values indicating more yellowness and negative values indicating more blueness [58]. In the context of orange juice, a higher positive value for b* reflects a more vibrant and intense yellow color, which is desirable and commonly associated with fresh and high-quality juice. The results indicated that the treatments involving the enzyme (for chitosan or silica supports) exhibit considerably lower b* values compared to the raw juice. This suggests a reduction in the yellowness of the juice when the enzyme is present during the clarification process. This reduction can be attributed to the hydrolysis of pectin. Pectinase hydrolyzes pectin, which is responsible for the structural integrity of cell walls. As a result, the cell contents, including carotenoids, are released [60,61].

An optimized clarification process has the potential to significantly enhance the color of orange juice through achieving an appropriate balance between turbidity reduction and maintaining the desired vibrant and vivid yellow appearance.

### 3.4. Analysis of Phenolic Compounds in Orange Juice

The phenolic compounds in orange juice and the treated samples were analyzed using HPLC-DAD at three different wavelengths, 280 nm, 320 nm, and 360 nm. The results are presented in Table 3, with the sum of the areas for each wavelength expressed as a percentage of the raw orange juice (considered as 100%).

In general, orange juices presents mainly flavones and flavanones linked to different substituents as phenolic compounds [62]. For all wavelengths, there is a statistically significant reduction in the phenolic compounds in the treated juices compared to the raw juices for most treatments, particularly when the supports were activated with genipin. In this case, for the samples treated with chitosan or silica activated with genipin, with or without the enzyme, the reduction in the phenolic compounds was statistically lower than other samples. Phenolic compounds, such as anthocyanins, proanthocyanidins, monomeric catechins, and phenolic acids, can interact with the chitosan–genipin matrix [63,64]; thus, their content in the treated juice could be lower compared to the raw sample. Comparing both matrices, when chitosan was used, it resulted in a higher preservation of the phenolic compounds compared to silica. However, when the matrices were modified with genipin or glutaraldehyde, there were no differences between chitosan and silica, indicating that the interactions between the phenolic compounds and activating agents or even the presence of the enzyme were more important. Similar results regarding enzymatic treatment were found in apple juice manufacturing processes. The authors observed a 19.9% reduction in the total procyanidin concentration in the juice through enzyme treatment [65]. In our study, using genipin-activated supports, the biocatalyst showed a 35% reduction in phenolic compounds, while for glutaraldehyde-activated matrices, the reduction was approximately 16%.

Overall, the results indicate that the choice of activating agent and support material (chitosan or silica) can influence the reduction of phenolic compounds, with potential preservation in specific cases. The presence of the enzyme did not alter, in general, the content of the phenolic compounds compared to the activated matrix, either with glutaraldehyde or genipin. However, the biocatalyst immobilized on glutaraldehyde-activated supports presented a higher phenolic content compared to the genipin-activated support. As mentioned before, this can be due to a higher reactivity and interaction between genipin and the phenolic compounds [63,64].

In a similar study, Benucci et al. [24] also observed that enzymatic treatment did not reduce the phenolic compounds indexes. Furthermore, the treated juices better preserved the anthocyanin pattern compared to the untreated juice over time [24].

### 3.5. Vitamin C

Vitamin C, also known as ascorbic acid, is an essential nutrient found in high concentrations in citrus fruits such as oranges [3]. It plays a crucial role in various physiological processes in the human body, including acting as an antioxidant, supporting the immune system, and aiding in the absorption of iron [4]. Therefore, the vitamin C content in orange juice is important. The content of ascorbic acid in the raw and treated juices was analyzed, and the results are presented in Table 4, expressed as a percentage of the raw orange juice (considered as 100%).

Looking at the results, vitamin C content was similar to raw orange juice for most of the treatments. The vitamin C content in all the treated samples remains relatively consistent, ranging from 82.0% to 105% of the vitamin C content in the raw juice, mostly being statistically equal to raw juice. This suggests that the treatment processes, including the use of chitosan or silica supports and activating agents, do not have a substantial impact on the degradation of vitamin C, since in most cases, especially using enzymes, the values were around 100 %. The exceptions were the treatments with CH-GLU, CH-GLU-E, and S-GEN. The consistency in vitamin C content across the samples is crucial for the quality of clarified orange juice. Vitamin C is a highly valued and sensitive nutrient that is susceptible to degradation during processing [66]. The fact that the treated samples exhibit similar vitamin C levels as the raw juice indicates that the chosen treatments have effectively preserved the vitamin C content. This is highly desirable as it ensures that the clarified orange juice retains its nutritional value and provides consumers with the expected health benefits associated with vitamin C consumption.

## 4. Conclusions

This study investigated the effects of different biocatalyst treatments on orange juice quality. Treatments involving chitosan and silica supports activated with genipin and glutaraldehyde, along with enzyme immobilization, significantly reduced turbidity compared to the raw juice. The highest turbidity reduction percentages were observed in CH-GEN-E and CH-GLU-E treatments, indicating their effectiveness in improving visual appearance. The soluble solids content (°Brix), pH, and reducing sugar concentration remained comparable to the raw juice, indicating minimal impact on these parameters. The analysis of phenolic compounds showed that samples treated with genipin activated supports presented significant alterations in composition. Importantly, the treated samples exhibited comparable vitamin C levels to the raw juice, preserving its nutritional value. These findings demonstrate the effectiveness of biocatalyst treatments in clarifying orange juice without compromising quality. Understanding the matrix effect in immobilized enzyme juice clarification is crucial for future process development.

## Figures and Tables

**Table 1:** Immobilization parameters for pectinase immobilized on chitosan and silica.

	Biocatalyst Activity (U·g^−1^)	IY (%)	IE (%)	RA (%)
Chitosan				
Genipin	1211.21 ± 22.56	30.37	27.3	8.3
Glutaraldehyde	535.17 ± 17.88	91.17	3.1	2.9
Silica				
Genipin	263.78 ± 7.23	20.12	44.02	8.86
Glutaraldehyde	152.21 ± 12.49	8.86	57.66	5.11

**Table 2:** Effects of different treatments on orange juice parameters.

Treatment	Turbidity Reduction (%)	TSS (°Brix)	Reducing Sugars (g·L^−1^)	pH	L*	a*	b*	C*
Orange juice	0.00 ^d^	10.2 ± 0.3 ^a^	5.20 ± 0.13 ^c,d^	3.47 ± 0.27 ^a^	68.81 ± 1.43 ^b^	−5.01 ± 0.03 ^b^	30.08 ± 1.22 ^a^	30.49
CH	3.67 ± 0.43 ^c,d^	9.3 ± 0.4 ^a,b,c^	5.30 ± 0.01 ^a,b,c,d^	3.39 ± 0.03 ^a^	63.95 ± 2.23 ^b^	−4.45 ± 0.42 ^b^	35.82 ± 0.31 ^a^	36.10
CH-GEN	5.56 ± 0.33 ^c,d^	8.9 ± 0.1 ^a,b,c^	5.40 ± 0.07 ^a,b,c,d^	3.37 ± 0.12 ^a^	63.65 ± 1.66 ^b^	−4.61 ± 0.21 ^b^	37.74 ± 0.88 ^a^	38.02
CH-GLU	6.14 ± 1.21 ^c^	10.1 ± 0.2 ^a^	5.17 ± 0.12 ^d^	3.36 ± 0.01 ^a^	66.5 ± 2.98 ^b^	−4.89 ± 0.55 ^b^	31.57 ± 0.92 ^a^	31.95
CH-GEN-E	93.57 ± 2.27 ^a^	9.0 ± 0.9 ^a,b,c^	5.21 ± 0.15 ^c,d^	3.36 ± 0.04 ^a^	78.93 ± 2.40 ^a^	−2.42 ± 0.12 ^a^	5.76 ± 0.25 ^b^	6.25
CH-GLU-E	94.15 ± 4.11 ^a^	9.4 ± 0.5 ^a,b^	5.51 ± 0.04 ^a,b^	3.45 ± 0.13 ^a^	78.6 ± 1.41 ^a^	−2.13 ± 0.15 ^a^	4.18 ± 0.13 ^b^	4.69
S	6.88 ± 0.66 ^c^	10.1 ± 0.3 ^a^	5.47 ± 0.03 ^a,b,c^	3.34 ± 0.11 ^a^	67.14 ± 1.83 ^b^	−4.96 ± 0.65 ^b^	33.94 ± 2.01 ^a^	34.30
S-GEN	8.61 ± 0.91 ^c^	8.5 ± 0.7 ^b,c^	5.34 ± 0.12 ^a,b,c,d^	3.27 ± 0.08 ^a^	63.68 ± 3.22 ^b^	−4.5 ± 0.31 ^b^	36.22 ± 0.33 ^a^	36.50
S-GLU	8.95 ± 0.87 ^c^	7.9 ± 0.4 ^c^	5.25 ± 0.11 ^b,c,d^	3.25 ± 0.02 ^a^	63.99 ± 1.89 ^b^	−4.57 ± 0.27 ^b^	35.7 ± 1.66 ^a^	35.99
S-GEN-E	89.33 ± 3.65 ^a^	8.0 ± 0.5 ^b,c^	5.40 ± 0.10 ^a,b,c,d^	3.28 ± 0.00 ^a^	78.43 ± 0.33 ^a^	−2.15 ± 0.12 ^a^	4.19 ± 0.72 ^b^	4.71
S-GLU-E	82.90 ± 2.13 ^b^	5.7 ± 0.5 ^d^	5.55 ± 0.07 ^a^	3.28 ± 0.14 ^a^	78.55 ± 1.54 ^a^	−2.66 ± 0.19 ^a^	7.22 ± 0.64 ^b^	7.69

Results are the mean ± standard deviation. Means followed by the same letters in the same columns do not differ according to Tukey’s test at 5% probability.

**Table 3:** Analysis of phenolic compounds, as percentages, at different wavelengths for the treatments on orange juice.

Treatment	280 nm	320 nm	360 nm	Mean
Orange juice	100.0 ^a^	100.0 ^a^	100.0 ^a^	100.0
CH	95.3 ± 3.4 ^a^	104.0 ± 2.1 ^a^	93.2 ± 1.1 ^b^	97.5
CH-GEN	62.4 ± 2.8 ^c^	76.6 ± 3.7 ^c^	63.5 ± 2.4 ^d^	67.5
CH-GLU	87.4 ± 1.5 ^b^	99.5 ± 5.4 ^a^	92.2 ± 4.7 ^b^	93.0
CH-GEN-E	59.4 ± 3.1 ^d^	67.3 ± 3.9 ^d^	59.8 ± 3.4 ^d^	62.2
CH-GLU-E	80.7 ± 2.9 ^b^	87.0 ± 4.4 ^b^	78.6 ± 2.8 ^c^	82.1
S	87.4 ± 4.4 ^b^	91.9 ± 1.8 ^b^	87.3 ± 2.5 ^b^	88.9
S-GEN	61.5 ± 1.2 ^c,d^	70.7 ± 2.3 ^c,d^	58.9 ± 5.1 ^d^	63.7
S-GLU	81.6 ± 3.9 ^b^	92.0 ± 1.6 ^b^	79.6 ± 4.4 ^c^	84.4
S-GEN-E	67.2 ± 4.1 ^c^	74.6 ± 3.1 ^c^	62.0 ± 3.6 ^d^	67.9
S-GLU-E	84.3 ± 2.0 ^b^	94.6 ± 3.4 ^a,b^	79.1 ± 2.5 ^c^	86.0

Results are the mean of three repetitions ± standard deviation. Means followed by the same letters in the same columns do not differ according to Tukey’s test at 5% probability.

**Table 4:** Analysis of vitamin C, as percentage, for the different treatments on orange juice.

Treatment	Vitamin C
Orange juice	100.0 ^a^
CH	101.0 ± 2.4 ^a^
CH-GEN	102.6 ± 1.6 ^a^
CH-GLU	82.0 ± 4.3 ^c^
CH-GEN-E	88.8 ± 2.8 ^b^
CH-GLU-E	102.5 ± 3.1 ^a^
S	105.0 ± 3.3 ^a^
S-GEN	90.5 ± 2.5 ^b^
S-GLU	103.1 ± 5.1 ^a^
S-GEN-E	103.0 ± 3.7 ^a^
S-GLU-E	102.0 ± 2.2 ^a^

Results are the mean of three repetitions ± standard deviation. Means followed by the same letters in the same columns do not differ according to Tukey’s test at 5% probability.

## Data Availability

The data used in this study are available upon request.
